# Differences in hierarchical structural changes between unoriented P(3HB) and P(3HB-*co*-3HH) under stretching

**DOI:** 10.1107/S1600576725002365

**Published:** 2025-04-25

**Authors:** Masato Arakawa, Taizo Kabe, Tadahisa Iwata, Mikihito Takenaka

**Affiliations:** a Institute for Chemical Research Kyoto University Gokasho Uji Kyoto611-0011 Japan; b Science of Polymeric Materials, Department of Biomaterial Sciences, Graduate School of Agricultural and Life Sciences University of Tokyo 1-1-1 Yayoi Bunkyo-Ku Tokyo113-8657 Japan; NSRRC, Taiwan

**Keywords:** P(3HB), P(3HB-*co*-3HH), crystalline polymers, USAXS, ultra-small-angle X-ray scattering, stretch-induced density fluctuations, *in situ*X-ray scattering

## Abstract

Poly[(*R*)-3-hy­droxy­butyrate] [P(3HB)] is a promising environmentally friendly polymer. However, P(3HB) exhibits brittle fracture, and the brittleness makes P(3HB) unsuitable for practical application. To improve the brittleness, 3-hy­droxy­hexano­ate (3HH) is introduced as a second monomer into P(3HB). The random copolymer P(3HB-*co*-3HH) shows a reduction in brittleness. In this paper, we investigate the changes in the hierarchical structures in unoriented P(3HB) and P(3HB-*co*-11mol%-3HH) during uniaxial stretching using *in situ* ultra-small-angle, small-angle and wide-angle X-ray scattering to clarify the effects of the introduction of 3HH. We reveal that the introduction of amorphous component units to P(3HB) not only suppresses crystallization and reduces crystal thickness on the nanometre scale but also destabilizes the crystal structure. We observed stretch-induced density fluctuations near the yield point by *in situ*X-ray scattering, showing heterogeneous deformation on scales from slightly larger than the lamellae to submicrometres in P(3HB) and on the submicrometre scale in its copolymer under uniaxial stretching. The enhancement of density fluctuations in the copolymer was smaller than that in P(3HB) because stress dispersion was caused by the hierarchical structure formed with the suppression of crystallization.

## Abstract 2

By using *in situ*X-ray scattering, the introduction of 3-hy­droxy­hexano­ate (3HH) to the eco-friendly polymer poly[(*R*)-3-hy­droxy­butyrate] [P(3HB)] was found to reduce both the density fluctuations on the submicrometre scale and the stability of the crystals, resulting in a reduction of the brittleness.

## 1. Introduction

Plastics have been used in numerous products because of their usability and processability. However, owing to their durability and stability, it is difficult to decompose plastic products in nature, and environmental pollution caused by plastic waste is a problem that needs urgent solution. There is also the problem of depletion of petroleum resources used for plastics and the CO_2_ emissions released during manufacturing. To overcome these problems, research on environmentally friendly polymers has been promoted in recent years. Poly[(*R*)-3-hy­droxy­butyrate] [P(3HB)] is a biodegradable polyester produced by microorganisms and is promising as an environmentally friendly polymer. Many researchers have studied P(3HB) for practical applications (Anderson & Dawes, 1990 ▸; Orts *et al.*, 1990 ▸; Sudesh *et al.*, 2000 ▸; Iwata, 2005 ▸; Iwata *et al.*, 2005 ▸; Kabe *et al.*, 2016 ▸).

P(3HB) has a fracture strength comparable to that of poly­propyl­ene. However, its fracture elongation is usually less than 5% (Anderson & Dawes, 1990 ▸; Sudesh *et al.*, 2000 ▸). More­over, secondary crystallization occurs at room temperature, resulting in the enhancement of brittleness (de Koning & Lem­stra, 1992 ▸; de Koning & Lemstra, 1993 ▸; Biddlestone *et al.*, 1996 ▸).

To improve the brittleness of P(3HB), copolymerization with a second monomer to reduce the crystallinity has been proposed (Liebergesell *et al.*, 1993 ▸; Sudesh *et al.*, 2000 ▸). The introduction of 3-hy­droxy­hexano­ate (3HH), an amorphous component monomer unit, is accomplished by microbial synthesis from palm oil. The produced random copolymer P(3HB-*co*-3HH) exhibits a crystallinity lower than P(3HB), and the ductility is improved.

P(3HB) is a crystalline polymer that forms a hierarchical structure similar to that of common polyolefins. The hierarchical structure is classified into four levels: crystal structures where molecular chains are folded regularly, lamella structures where amorphous and crystalline phases are alternately stacked, fibril structures developed by the growth of lamellae, and spherulite structures formed by the filling of fibrils.

Many researchers have investigated the correlation between the internal structure and physical properties of oriented P(3HB) and its copolymers, and the structural changes during uniaxial stretching. However, there are only a few cases in which the structural changes during uniaxial stretching of unoriented samples have been investigated and the correlations with physical properties have been clarified. Moreover, the changes in the hierarchical structures on the submicrometre scale have not been investigated, although the structures on the submicrometre scale strongly correlate to the mechanical properties described below.

The structures of crystalline polymers have been widely studied by small- and wide-angle X-ray scattering (SAXS and WAXS, respectively). When a crystalline polymer is uniaxially stretched, the strain–stress (S–S) curve reflects the strength and ductility of the material, and the yield behavior on the S–S curve reflects the change in internal structure. Therefore, changes in lamellar and crystalline structures under uniaxial drawing have been investigated by *in situ* SAXS and WAXS (Butler *et al.*, 1997 ▸; Sakurai *et al.*, 2005 ▸; Guo *et al.*, 2015 ▸).

The recent development of ultra-small-angle scattering (USAXS) has also enabled the study of the correlation between structure and physical properties on the submicrometre scale, on the order of 100 nm. USAXS observation of the crystalline polymer polyethyl­ene provides information on the fibril structures, which are larger than the lamellar structures. In our previous study (Takenaka *et al.*, 2007 ▸; Kishimoto *et al.*, 2020 ▸) using USAXS, we observed an ‘abnormal’ butterfly pattern in which the scattering intensity parallel to the direction of elongation increases near the yield point during uniaxial tensile testing of polyethyl­ene. The abnormal butterfly pattern has been observed in swollen gels (Bastide *et al.*, 1990 ▸), slide-ring gels under uniaxial drawing (Karino *et al.*, 2005 ▸), and stretched mixtures of short and long polystyrene homopolymers (Hayes *et al.*, 1996 ▸). The increase in scattering intensity along the deformation direction indicates an enhancement of density or concentration fluctuations induced along the same direction during stretching. The stretch-induced density or concentration fluctuations are driven by spatial inhomogeneity of deformation resulting from the difference in deformation amounts between regions with disparate mechanical properties. Doi & Onuki (1992 ▸), Furukawa & Tanaka (2009 ▸) and Kurotani & Tanaka (2022 ▸) proved theoretically that shear-induced enhancement in density fluctuations results from the gradient of the stress field caused by the spatial asymmetry of mechanical properties associated with the density field. Thus, the abnormal butterfly pattern observed during the drawing of polyethyl­ene reflects the difference in deformation between fibril regions with high crystallinity and non-fibril regions with low crystallinity, as crystalline polymers have spatially heterogeneous stress fields associated with crystallinity fluctuations.

In this study, we investigated the structural changes of unoriented P(3HB) and P(3HB-*co*-3HH) under uniaxial stretching in a time-resolved manner over a wide *q*-range by USAXS, SAXS and WAXS. By comparing the two, we show how the introduction of the copolymer P(3HH) affected the changes in hierarchical structures of P(3HB) and P(3HB-*co*-3HH) under uniaxial stretching and clarify the origin of the improvement of the mechanical properties.

## 2. Experiment

### 2.1. Sample preparation

We used P(3HB) and P(3HB-*co*-11mol%-3HH) samples provided by the Iwata laboratory, University of Tokyo.

Sheets of each material were prepared using the following steps. Firstly, we pressurized each material in a 0.5 mm-thick mold for 10 min to melt it with a press machine set at 180°C. The melt was then immediately transferred to a vacuum oven set at 60°C and quenched for over 2 h to produce a 0.5 mm-thick pressed sheet. Finally, dumbbell specimens with a center width of 3.2 mm were punched from the sheets.

### 2.2. *In situ*X-ray scattering measurements

We performed *in situ*X-ray scattering (USAXS/SAXS/WAXS) measurements during uniaxial stretching in the second hutch of beamline BL03XU at SPring-8, Hyogo, Japan (Masunaga *et al.*, 2011 ▸). Using a custom-made tensile testing machine designed to measure stress–strain curves and X-ray scattering simultaneously, dumbbell specimens were stretched at 1 mm min^−1^ at room temperature.

We performed USAXS measurements with an X-ray exposure time of 400 ms. The incident X-ray wavelength, the specimen-to-detector length and the beam size at the center of specimens were 2.0 Å, 7.84 m and 160 µm (horizontal) × 80 µm (vertical), respectively. The detector was a PILATUS-1M (Dectris Ltd). In addition, as attenuators, a 100 µm-thick Al plate for P(3HB) and a 200 µm-thick plate for P(3HB-*co*-11mol%-3HH) were used.

The simultaneous SAXS and WAXS measurements were performed with exposure times of 400 ms for P(3HB) and 800 ms for P(3HB-*co*-11mol%-3HH). The incident X-ray wavelength was 1.0 Å and the beam size at the center of the specimens was 300 µm (horizontal) × 300 µm (vertical). A 10 µm-thick Au plate was used as the attenuator. The camera length was 2.35 m/105 mm and a PILATUS detector was used for the SAXS/WAXS measurements.

We corrected the 2D scattering images for the transmittances of X-rays and subtracted air and background scattering from them for further analyses. The data obtained by the USAXS measurement for P(3HB-*co*-11mol%-3HH) were accumulated from two consecutive datasets to make a single dataset.

## 3. Results and discussion

### 3.1. S–S curve

**Figure 1:**
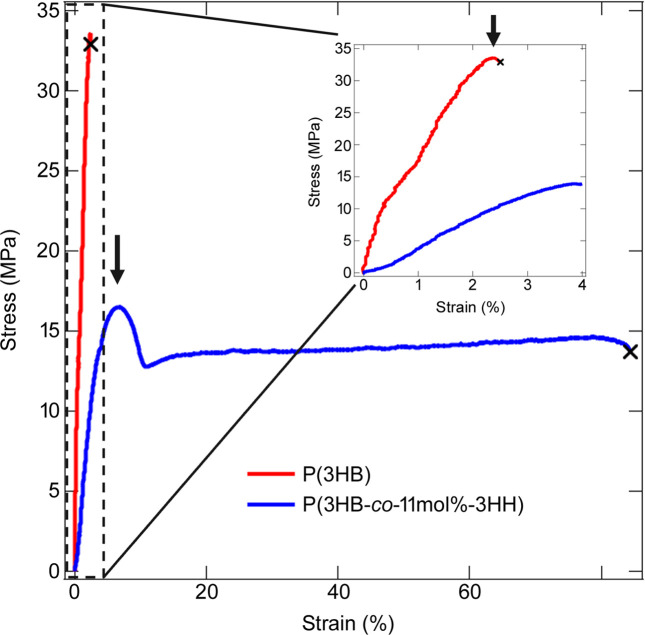
S–S curves of P(3HB) and P(3HB-*co*-11mol%-3HH). Arrows indicate the yield points and crosses indicate the elongations at break.

Fig. 1 ▸ shows S–S curves obtained during the simultaneous SAXS and WAXS measurements. The stress is the engineering stress and the strain ɛ is defined by the following equation:
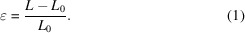
Here, *L* and *L*_0_ are the chuck distances before and after stretching, respectively.

The yield points for P(3HB) and P(3HB-*co*-11mol%-3HH) were observed at ɛ = 2.38 and 6.74%, respectively. Although Young’s modulus of the copolymer was lower than that of the homopolymer, the introduction of the amorphous monomer units improved the breaking elongation as shown in Fig. 1 ▸.

### 3.2. Comparison of the hierarchical structures of unstretched P(3HB) and P(3HB-*co*-11mol%-3HH)

**Figure 2:**
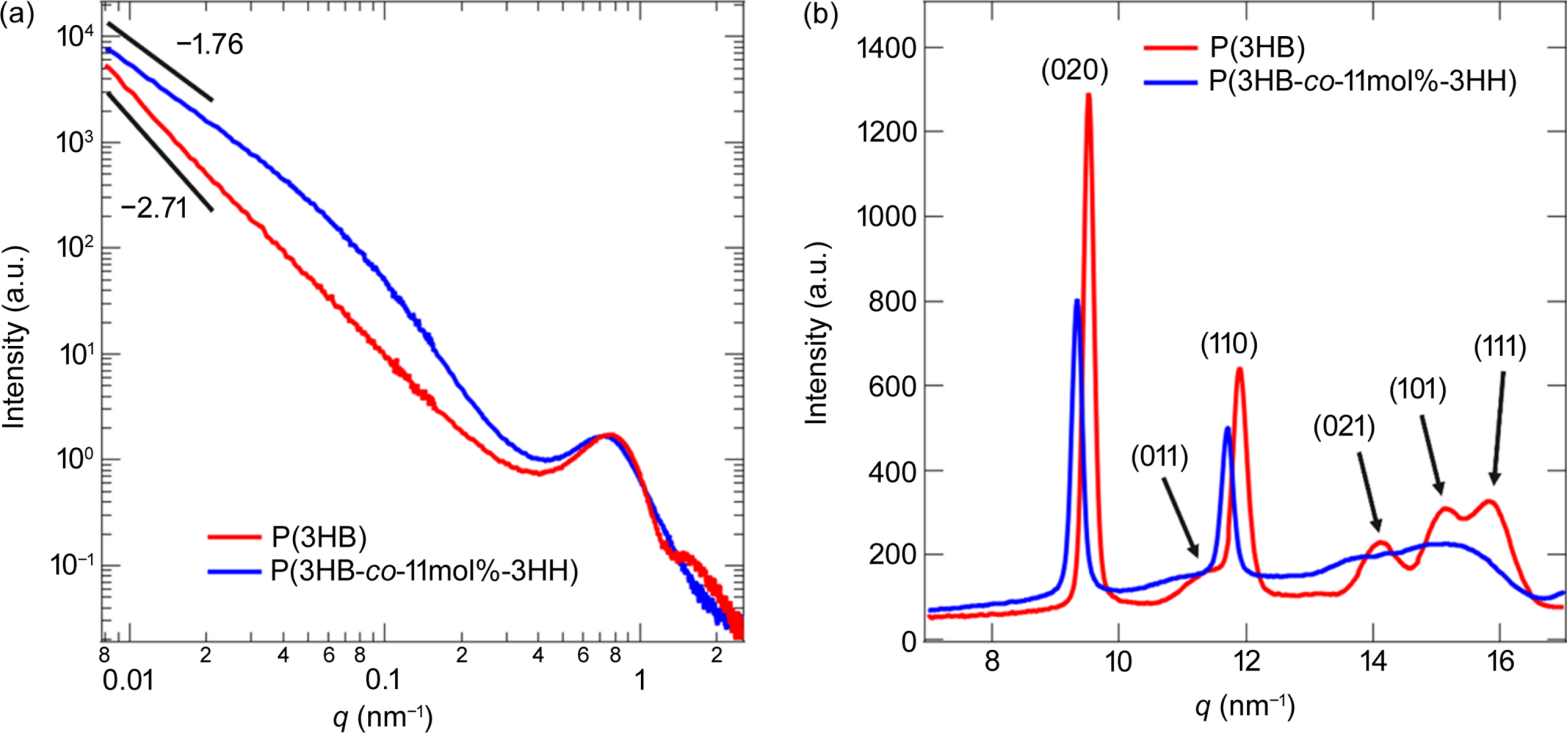
(*a*) Combined USAXS/SAXS 1D profiles and (*b*) WAXS profiles of P(3HB) and P(3HB-*co*-11mol%-3HH) before stretching (ɛ = 0%).

**Figure 3:**
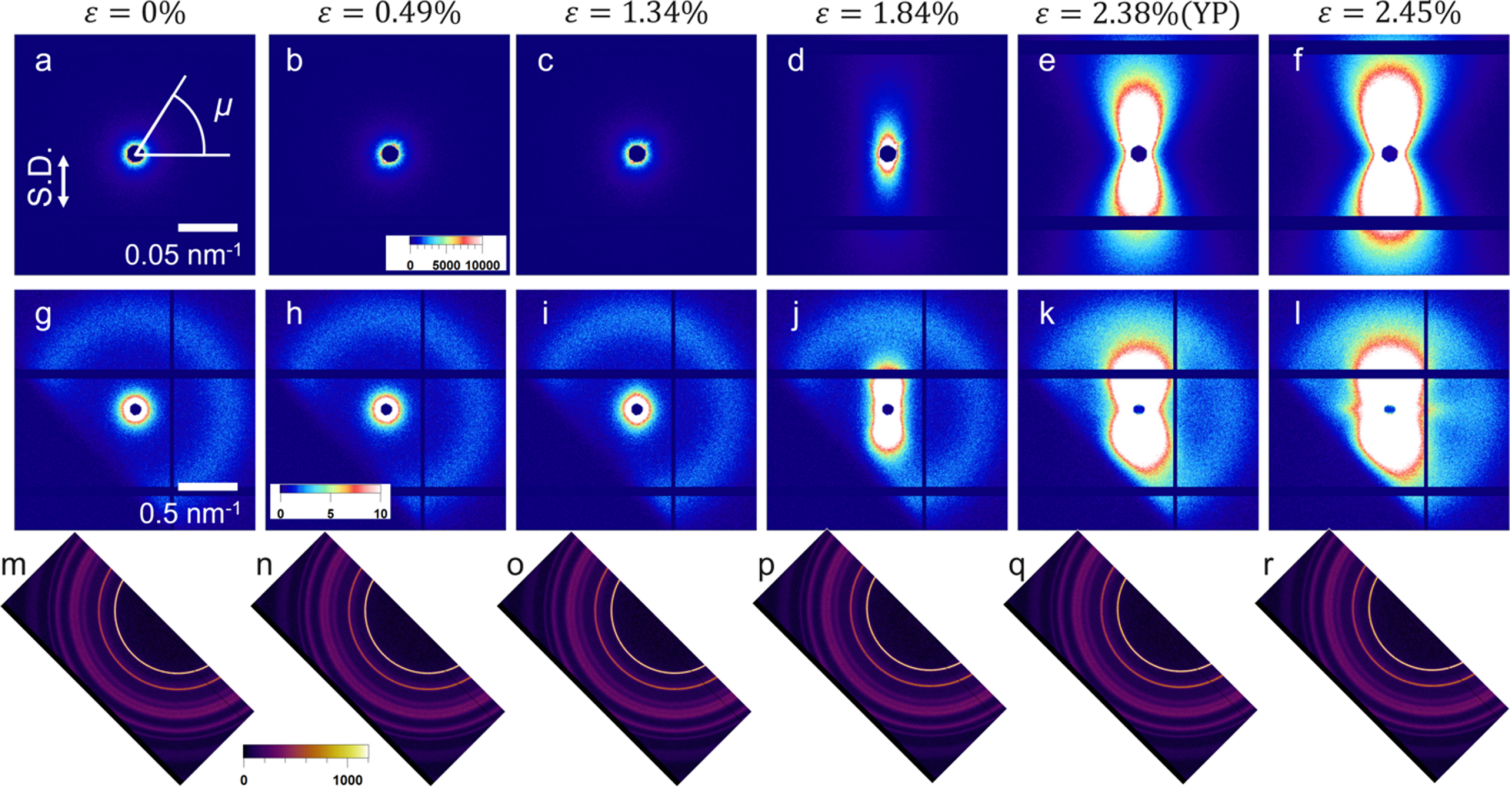
(*a*)–(*f*) USAXS, (*g*)–(*l*) SAXS and (*m*)–(*r*) WAXS 2D patterns of P(3HB) with strain. The stretching direction (S.D.) is parallel to the arrow in (*a*).

Fig. 2 ▸ shows the scattering intensity *I*(*q*) of USAXS, SAXS and WAXS for P(3HB) and P(3HB-*co*-11mol%-3HH) before stretching. Here, *q* is the magnitude of the scattering vector **q** and is given by *q* = (4π/λ)sin(θ/2), where λ and θ are the wavelength of the incident X-rays and the scattering angle, respectively. Note that the USAXS, SAXS and WAXS 2D patterns [Figs. 3(*a*), 3(*g*) and 3(*m*) for unstretched P(3HB) and Figs. 8(*a*), 8(*g*) and 8(*m*) for unstretched P(3HB-*co*-11mol%-3HH)] were isotropic. The WAXS peaks of the copolymer were located at lower *q* values than those of the homopolymer because the crystal structure was changed slightly by introducing amorphous component monomer units, as reported by Doi *et al.* (1995 ▸), indicating that the addition of the comonomer prevents the chain from folding neatly. Moreover, the homopolymer profile clearly showed higher-order peaks, whereas the copolymer profile was less distinct, indicating the lack of long-range order of the crystal structure of the co­poly­mer.

We found power-law behaviors of P(3HB) and the co­poly­mer with the exponents being 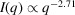 and 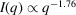, respectively, at 0.008 < *q* < 0.02 nm^−1^, as shown in Fig. 2 ▸(*a*). Fig. 2 ▸(*a*) demonstrates that the USAXS intensity of the copolymer was larger than that of the homopolymer when unstretched, indicating that the density fluctuations of the copolymer on the submicrometre scale were larger.

At *q* > 0.02 nm^−1^, the scattering intensity reflects the lamellar structures. A higher-order peak was seen in the scattered intensity of P(3HB), whereas the scattering intensity of P(3HB-*co*-11mol%-3HH) did not show the higher-order peak, suggesting that the regularity of the stacking of the homopolymer was higher than that of the copolymer. To characterize the lamellar structures, we calculated correlation functions (Strobl & Schneider, 1980 ▸; Strobl *et al.*, 1980 ▸) from the scattering profiles for both specimens. The details of the calculation for the correlation function are described in the supporting information. The *d*-spacing (*d*_l_) and crystal thickness (*d*_c_) of each specimen were estimated as listed in Table 1 ▸ by comparing the crystallinity obtained from WAXS, discussed below, with the ratio of the thickness to the *d*-spacing. The *d*-spacing in the copolymer was larger than that of the homopolymer and the crystal thickness was smaller. The reduction of crystal thickness in the copolymer was attributed to the suppression of crystallization of P(3HB) by the introduction of amorphous component monomer units.

Finally, we determined the crystallinity of each sample by fitting the WAXS 1D profile with Gaussian functions and a constant background to model the peak intensities of the crystalline peaks and an amorphous halo. The parameters obtained are listed in Table 1 ▸. The crystallinity of the copolymer was lower than that of the homopolymer, and this trend is consistent with the results of SAXS analysis.

In summary, P(3HB) had high crystallinity, and the spherulites exhibited a high regularity of lamellar stacking. On the other hand, P(3HB-*co*-11mol%-3HH) had low crystallinity, and fibrils with a lower regularity of lamellar stacking filled the spherulites.

### 3.3. Changes in the hierarchical structure of P(3HB) under stretching

**Figure 4:**
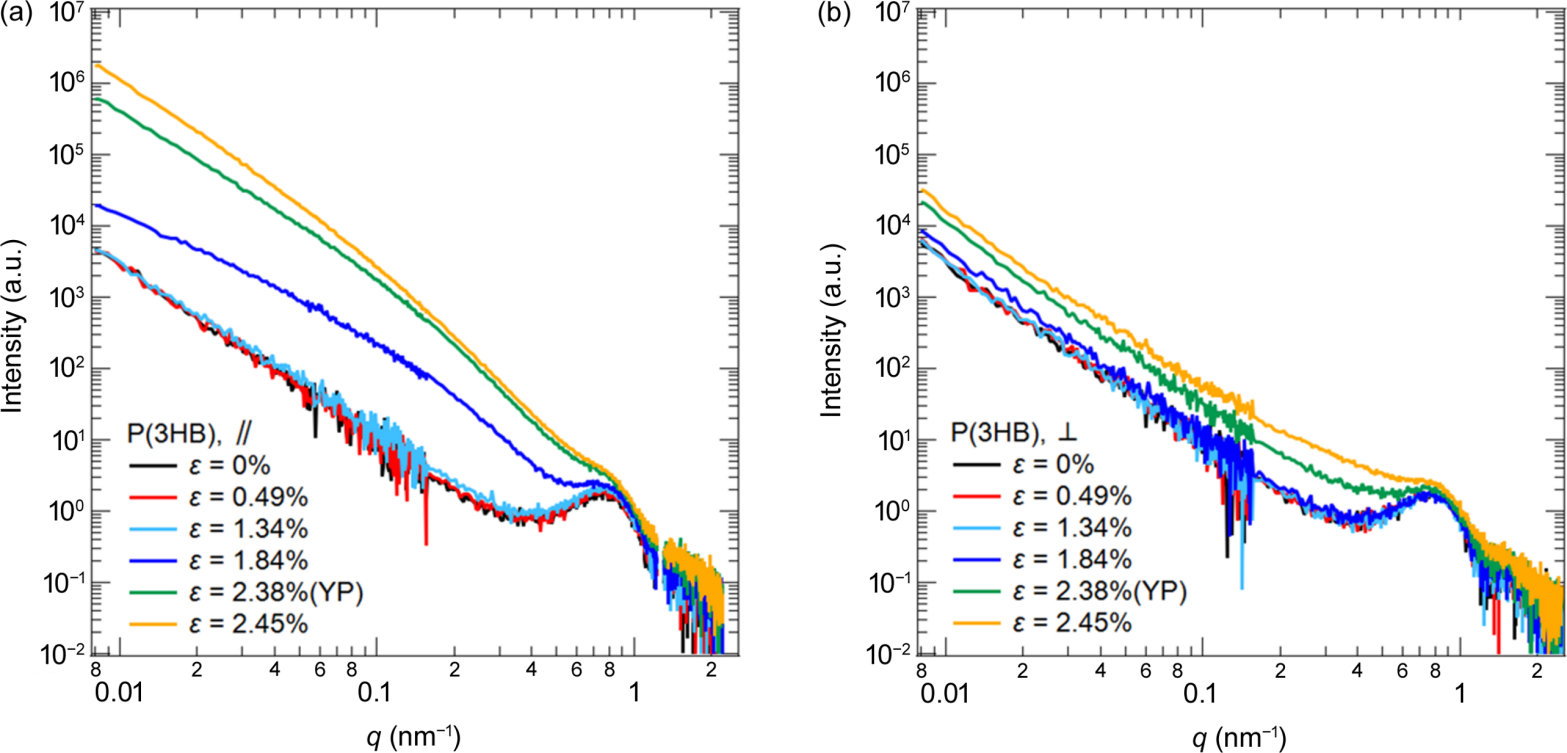
Combined USAXS and SAXS profiles for P(3HB). The profiles in (*a*) and (*b*) correspond to 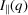 and 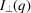, respectively.

Fig. 3 ▸ shows the changes in the USAXS, SAXS and WAXS 2D patterns with strain for the P(3HB) homopolymer, and Fig. 4 ▸ shows the combined USAXS and SAXS 1D profiles along the directions parallel [denoted 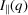] and perpendicular [denoted 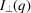] to the stretching direction. We obtained the 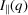 and 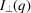 profiles from the USAXS and SAXS 2D patterns by averaging the sector within ±2.5° from the stretching direction (μ = 90°) and its vertical (μ = 0°), respectively. Azimuthal angle μ is defined in Fig. 3 ▸.

Near the yield point (YP), inhomogeneous deformation occurred on the submicrometre scale in P(3HB). After ɛ = 1.8%, 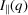 increased significantly in the USAXS region, as shown in Fig. 4 ▸, and the abnormal butterfly patterns are observed clearly in Fig. 3 ▸. These patterns in USAXS represent heterogeneous deformations on the submicrometre scale under uniaxial stretching, and density fluctuations are induced by uniaxial stretching. Kishimoto *et al.* (2020 ▸) observed the abnormal butterfly patterns in polyethyl­ene under uniaxial stretching. As Kishimoto *et al.* (2020 ▸) suggested, the spatial inhomogeneity of the stress field arising from the inhomogeneity of the crystallinity causes strain-induced density fluctuations. Furukawa & Tanaka (2009 ▸) and Kurotani & Tanaka (2022 ▸) have theoretically indicated that a spatial inhomogeneity in the stress field enhances the concentration or density fluctuation during deformation.

As shown in Fig. 3 ▸, scattering patterns in the SAXS like the abnormal butterfly patterns observed in the USAXS were also observed near the yield point. The peaks from the lamellar structures were overlapped by these patterns. These patterns indicate that heterogeneous deformation also occurred on length scales slightly larger than the lamellae.

After the yield point, weak streak scattering perpendicular to the stretching direction appeared, and a slight enhancement in 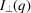 was observed in Figs. 3 ▸(*l*) and 4 ▸(*b*). This streak scattering reflected the formation of string-like nanovoids aligned to the strain direction. The voids originate from the density fluctuations enhanced by stretching.

**Figure 5:**
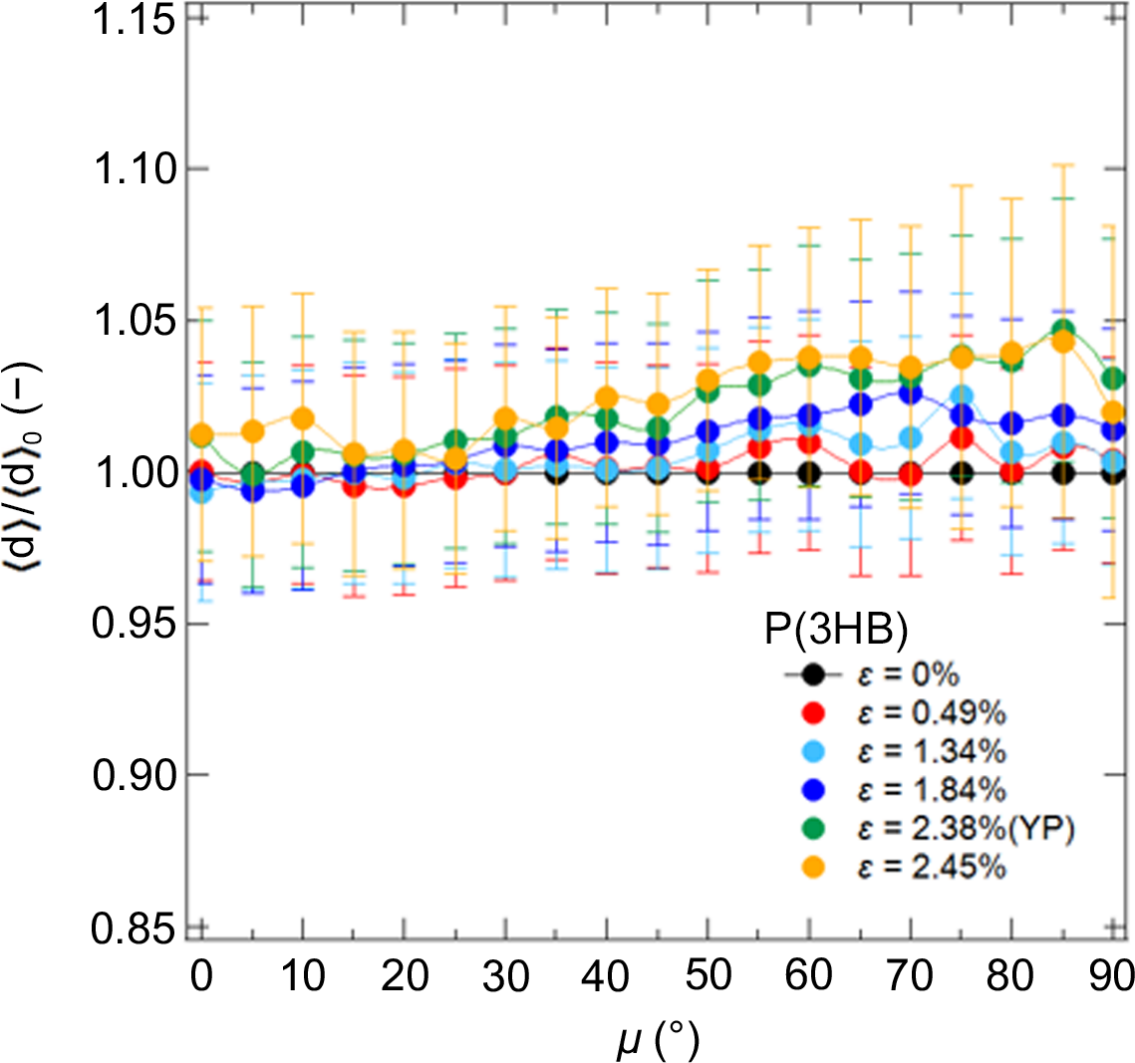
μ dependence of 〈*d*〉/〈*d*〉_0_ for P(3HB) with strain.

To investigate the changes in lamellar structure caused by stretching in more detail, we calculated the correlation functions (Strobl & Schneider, 1980 ▸; Strobl *et al.*, 1980 ▸; Hashimoto, 2022 ▸) from the SAXS 1D profiles obtained by averaging sectors in 2D patterns at every 5° from μ = 0 to μ = 90°. We estimated the average of *d*-spacing, 〈*d*〉, from the correlation functions and calculated 〈*d*〉/〈*d*〉_0_ at each μ as shown in Fig. 5 ▸. 〈*d*〉_0_ is the 〈*d*〉 at ɛ = 0%. Fig. 5 ▸ shows that, as the stretch ratio increases, 〈*d*〉/〈*d*〉_0_ along μ = 90° tends to increase.

**Figure 6:**
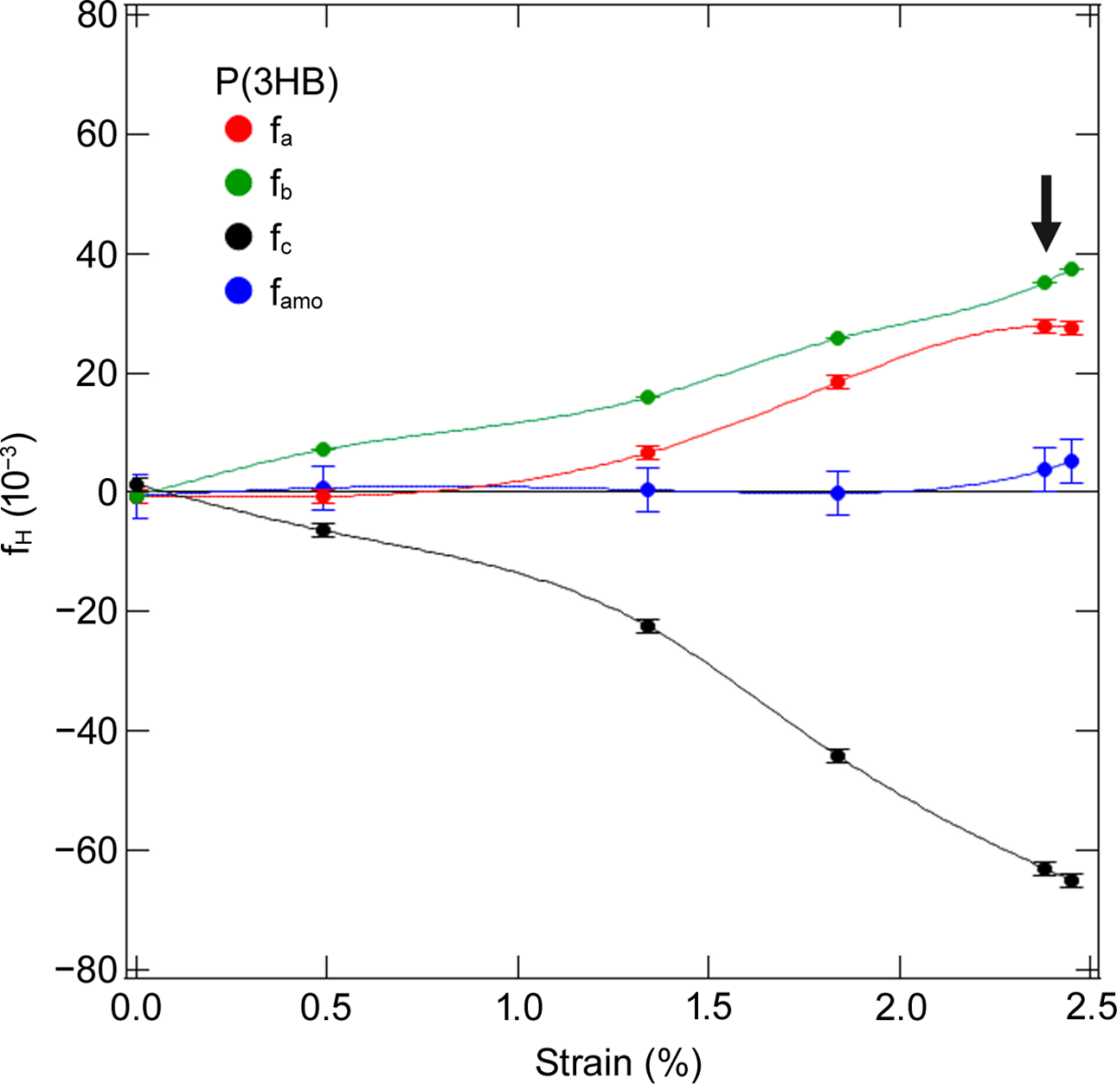
Changes in *f*_H_ for P(3HB) with strain. Arrow indicates the yield point.

Furthermore, we analyzed the WAXS 1D profiles and calculated the Hermans orientation factor (*f*_H_) of the *a*, *b* and *c* axes (polymer chain) of the crystal. We obtained the WAXS 1D profiles sector-averaged at every 5° from μ = 180° to μ = 270° from the 2D patterns. The orientation factor is defined as
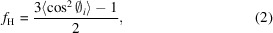
where 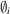 is the angle between the *a*, *b* or *c* axis and the stretching direction. 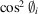 is defined by equations in the supporting information. We obtained the orientations of the *a*, *b* and *c* axes (*f_a_*, *f_b_*, *f_c_*) and estimated the orientation information of the amorphous polymer chains (*f*_amo_) using Wilchinsky’s method (1959 ▸, 1960 ▸). *f*_H_ = 1 represets perfect parallel alignment, whereas the value is −0.5 for perfect perpendicular alignment. *f*_H_ = 0 represents random orientation or the orientation along the magic angle (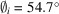). The ɛ dependences of *f_a_*, *f_b_*, *f_c_* and *f*_amo_ for the homopolymer are shown in Fig. 6 ▸. These results reveal that the *c* axis of the crystal was aligned perpendicularly to the direction of stretching, while the *a* and *b* axes were oriented parallel. In addition, polymer chains in amorphous phases were oriented slightly along the stretching direction.

**Figure 7:**
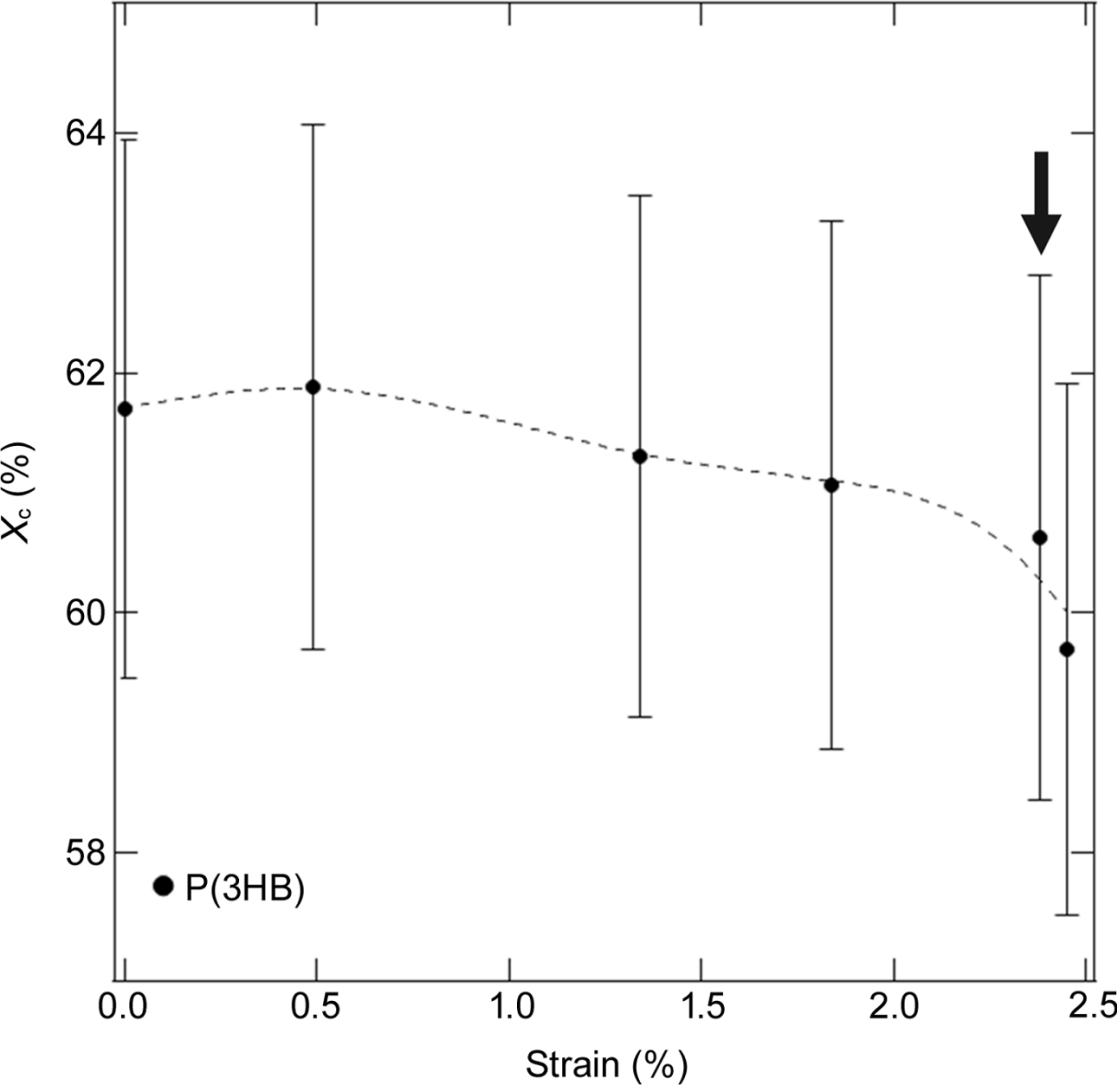
Crystallinity of P(3HB) at each ratio. Arrow indicates the yield point.

We also estimated the crystallinity of the homopolymer at each stretch ratio from the peak separation of the WAXS 1D profiles using Gaussian functions and a constant background, as shown in Fig. 7 ▸. The crystallinity decreased during stretching and significantly declined after the yield point. Considering the collapse of the lamellar structure parallel to the stretching direction with strain, the change in crystallinity indicates that mechanical melting occurred by the coarse slip in the lamellae after the yield point, as seen in polyethyl­ene (Gerasimov *et al.*, 1974 ▸; Butler *et al.*, 1997 ▸; Kishimoto *et al.*, 2020 ▸). Coarse slip represents the fragmentation of the crystalline parts in the lamellae into smaller blocks, leading to lamellar disintegration. The collapse of the lamellar structure is discussed in the supporting information.

### 3.4. Changes in the hierarchical structure of P(3HB-*co*-11mol%-3HH) under stretching

**Figure 8:**
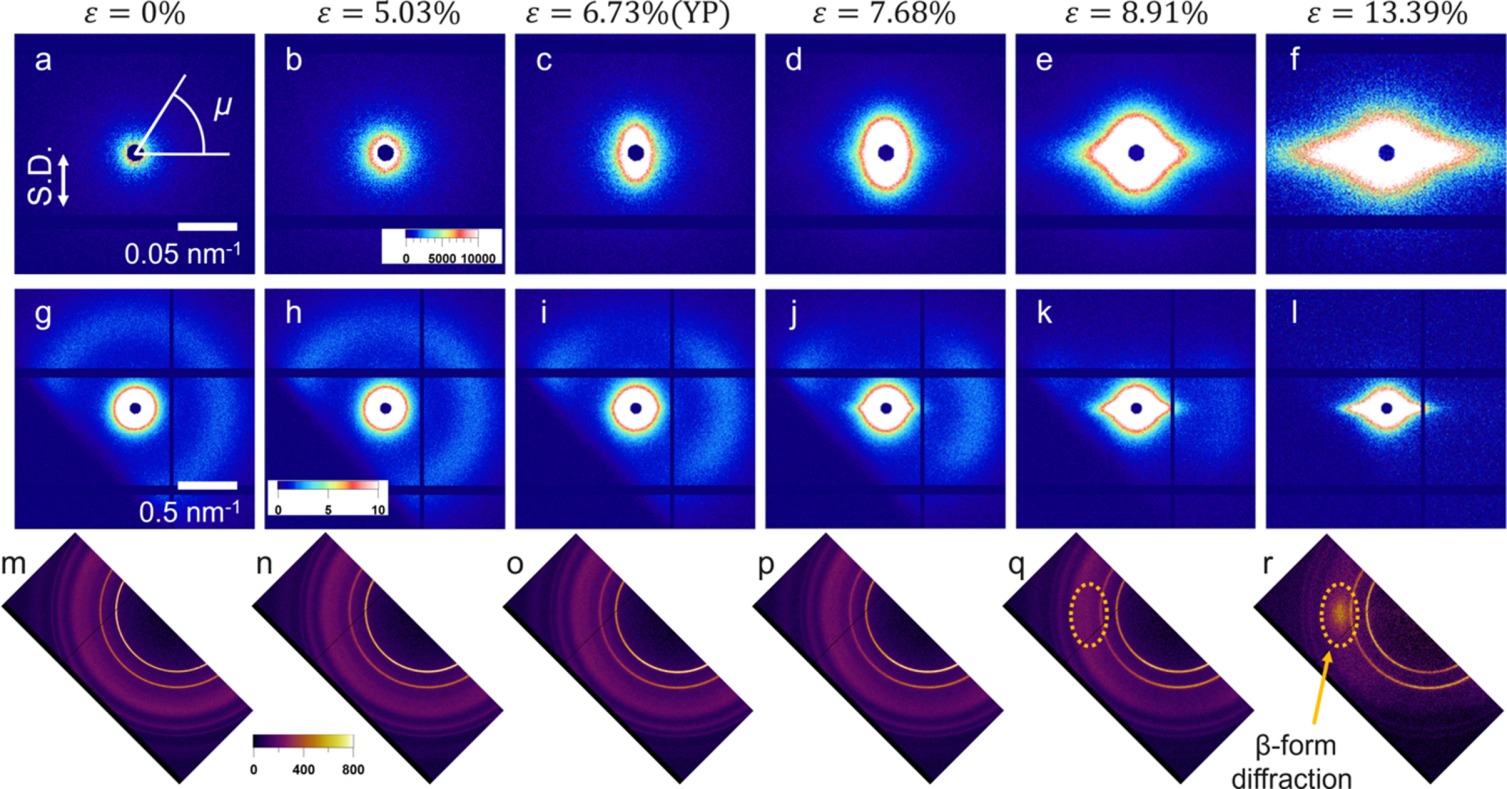
(*a*)–(*f*) USAXS, (*g*)–(*l*) SAXS and (*m*)–(*r*) WAXS 2D patterns of P(3HB-*co*-11mol%-3HH) with strain. S.D. is parallel to the arrow in (*a*).

**Figure 9:**
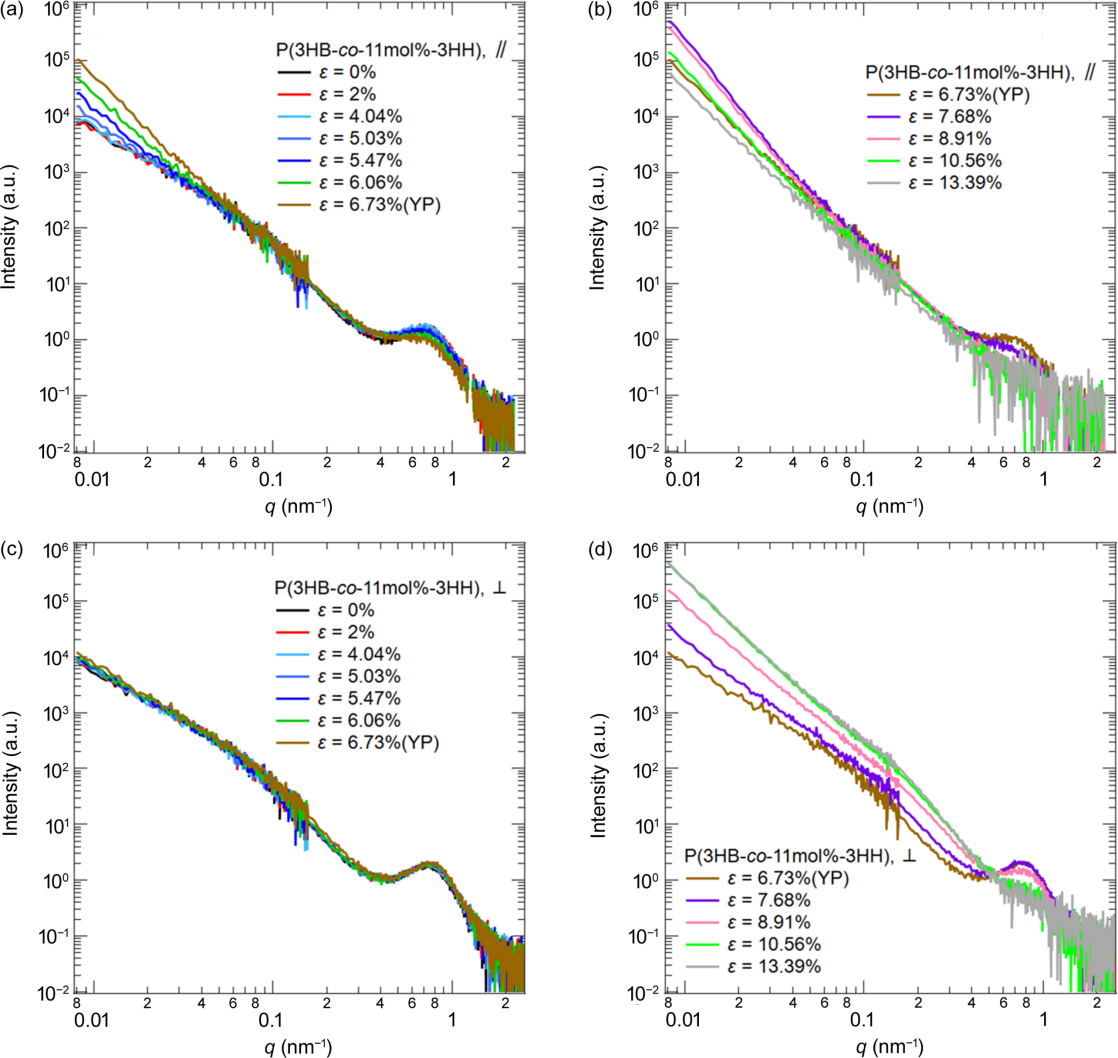
Combined USAXS and SAXS profiles for P(3HB-*co*-11mol%-3HH). The profiles in (*a*) and (*b*) correspond to 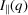, and those in (*c*) and (*d*) correspond to 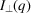.

Fig. 8 ▸ shows the changes in the USAXS, SAXS and WAXS 2D patterns with strain for P(3HB-*co*-11mol%-3HH), and Fig. 9 ▸ shows the combined USAXS and SAXS 1D profiles, 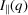 and 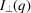. Scattering patterns at ɛ not shown in Fig. 8 ▸ are provided in the supporting information.

**Figure 10:**
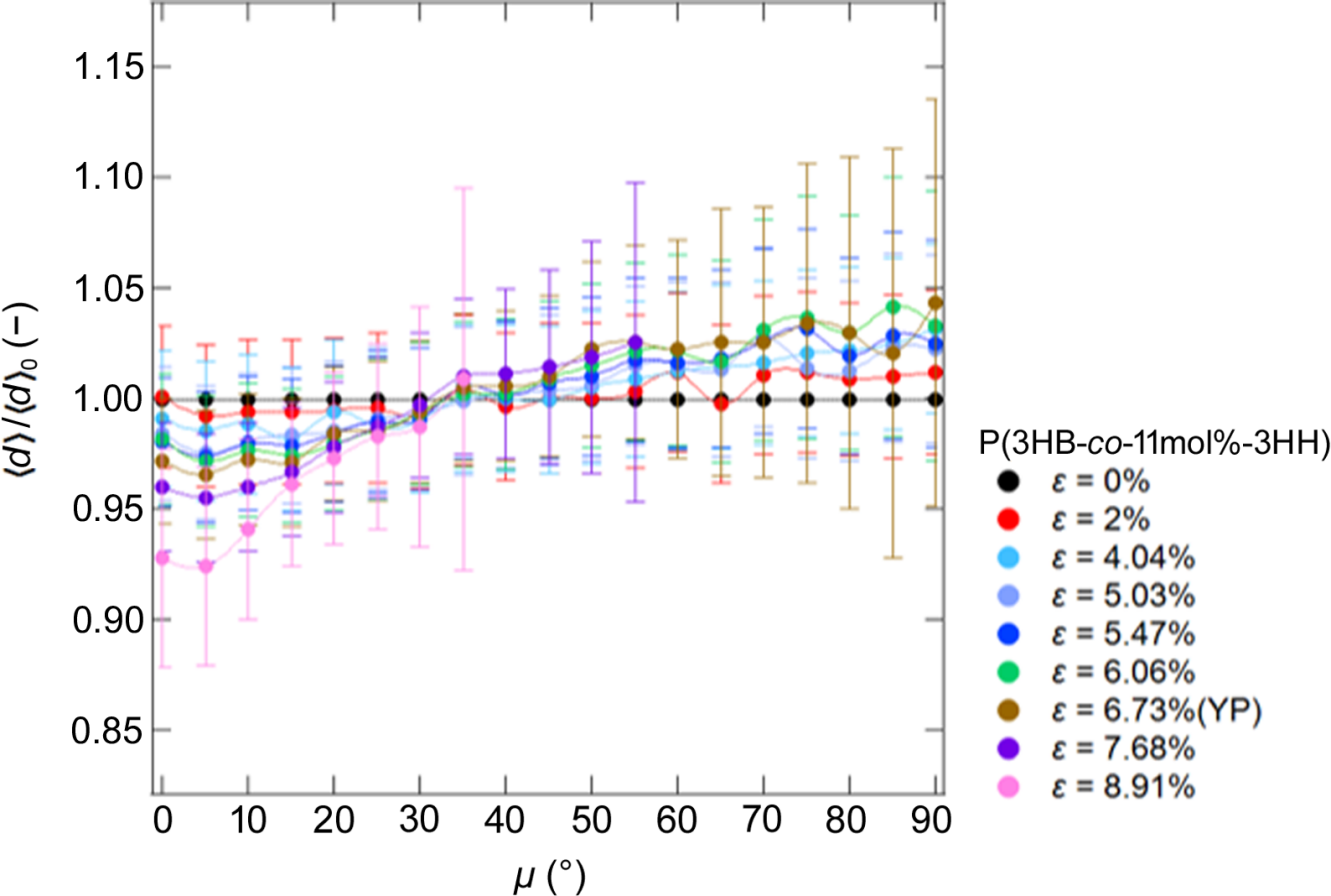
μ dependence of 〈*d*〉/〈*d*〉_0_ for P(3HB-*co*-11mol%-3HH) with strain.

Similar to the case of P(3HB), an increase in 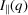 in the USAXS patterns was observed. However, no clear abnormal butterfly pattern was observed in the USAXS in P(3HB-*co*-11mol%-3HH). This is because the spatial inhomogeneity of the stress field is lower than that of the homopolymer. As shown in the WAXS profiles at ɛ = 0% in Fig. 2 ▸(*b*), the long-range order of the crystal structure of the copolymer was poorer than that of the homopolymer. Due to the poor long-range order in the copolymer, the stress of the higher-crystallinity region or the fibrils for the copolymer was lower than that for the homopolymer. Thus, the inhomogeneity of the stress field for the copolymer was lower than that for the homopolymer. We calculated the change in μ dependence of 〈*d*〉/〈*d*〉_0_ with strain and the ɛ dependences of orientation factors and crystallinity for the copolymer as shown in Figs. 10 ▸, 11 ▸ and 12 ▸, respectively. The changes in the parameters with strain clarified that the changes in the hierarchical structures with stretching are divided into region I [0 ≤ ɛ ≤ 6.73% (the yield point)] and region II (7.68% ≤ ɛ). The features of each region are shown below.

#### 3.4.1. Region I [0 ≤ ɛ ≤ 6.73% (the yield point)]

Clear abnormal butterfly patterns for the copolymer were not observed in USAXS near the yield point, unlike the case of the homopolymer, as shown in Fig. 8 ▸. The enhancement in 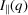 was less than that of the homopolymer. This indicates that the reduction of the stability of the crystals by introducing the amorphous component monomer unit suppressed spatial heterogeneity of the stress field between fibrillar and non-fibrillar regions more than in the homopolymer, improving the deformation heterogeneity on the submicrometre scale. A discussion of the reduction in crystal stability is included in the supporting information.

As shown in Fig. 8 ▸, we observed the disappearance of the SAXS peaks along μ = 90° after ɛ = 5.03%, indicating that the stacking of lamellae along the stretching direction was deformed by the coarse slip, as seen in polyethyl­ene (Gerasimov *et al.*, 1974 ▸; Butler *et al.*, 1997 ▸; Kishimoto *et al.*, 2020 ▸).

According to the result from the correlation functions, the average of the *d*-spacing in the lamellar structures, 〈*d*〉/〈*d*〉_0_, at μ = 0° decreased as the copolymer was elongated. In addition, 〈*d*〉/〈*d*〉_0_ at μ = 90° increased from ɛ = 0% to ɛ = 4.04% but did not change much after ɛ = 5.03% from the value at ɛ = 4.04%. This indicates that the copolymer elongated in the direction parallel to the stretching direction and contracted in the perpendicular direction in accordance with the stretching ratio until ɛ = 4.04%. By contrast, after ɛ = 5.03%, a slight coarse slip occurred in the stacked lamellae, resulting in little change in 〈*d*〉/〈*d*〉_0_ along the strain direction.

**Figure 11:**
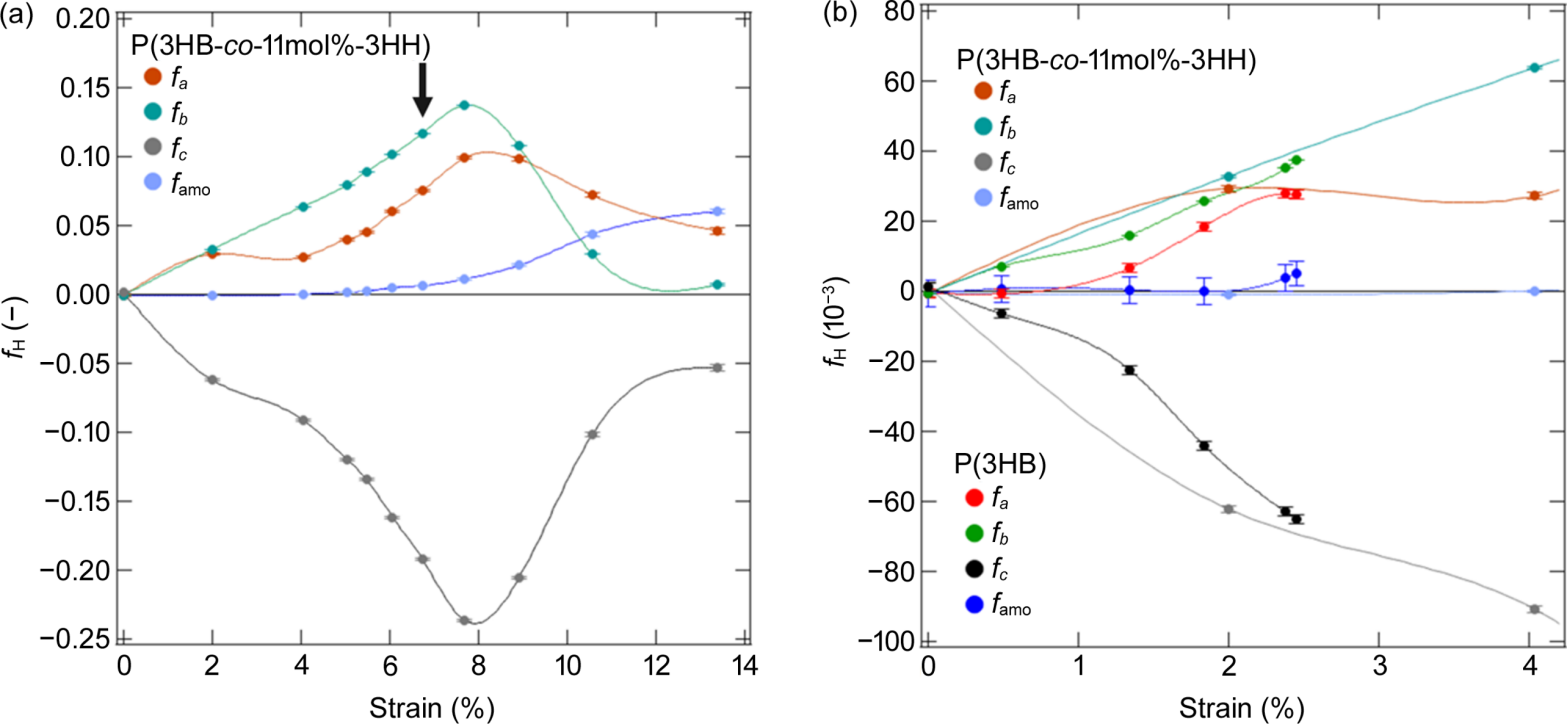
(*a*) Changes in *f*_H_ for P(3HB-*co*-11mol%-3HH) with strain and (*b*) comparison of *f*_H_ for P(3HB) and P(3HB-*co*-11mol%-3HH). Arrow in (*a*) indicates the yield point of P(3HB-*co*-11mol%-3HH).

As shown in Fig. 11 ▸(*b*), the ɛ dependences of *f_a_*, *f_b_*, *f_c_* and *f*_amo_ for the copolymer were in good agreement with the *f*_H_ changes for the homopolymer up to rupture. This indicates that the introduction of P(3HH) does not affect the degree of orientation of the polymer chains and P(3HB) crystals under stretching. Given the large difference in stress between the homopolymer and the copolymer under stretching, note that the energy required to orient the crystals in a particular direction is lower for the copolymer. The effect of copolymerization is mainly in the suppression of crystallization and the dispersion of stress due to the hierarchical structure formed with the suppression. After ɛ = 4.04%, where the homopolymer broke, the *c* axis of the crystals was oriented even closer to perpendicular to the stretching direction, and the *a* axis, *b* axis and amorphous polymer chains were oriented even closer to parallel, as shown in Fig. 11 ▸(*a*). The degrees of changes in *f_a_*, *f_b_*, *f_c_* and *f*_amo_ were slightly greater after ɛ = 5.03%. This indicates that coarse slip in the lamellae facilitated the orientations of crystals and amorphous chains.

**Figure 12:**
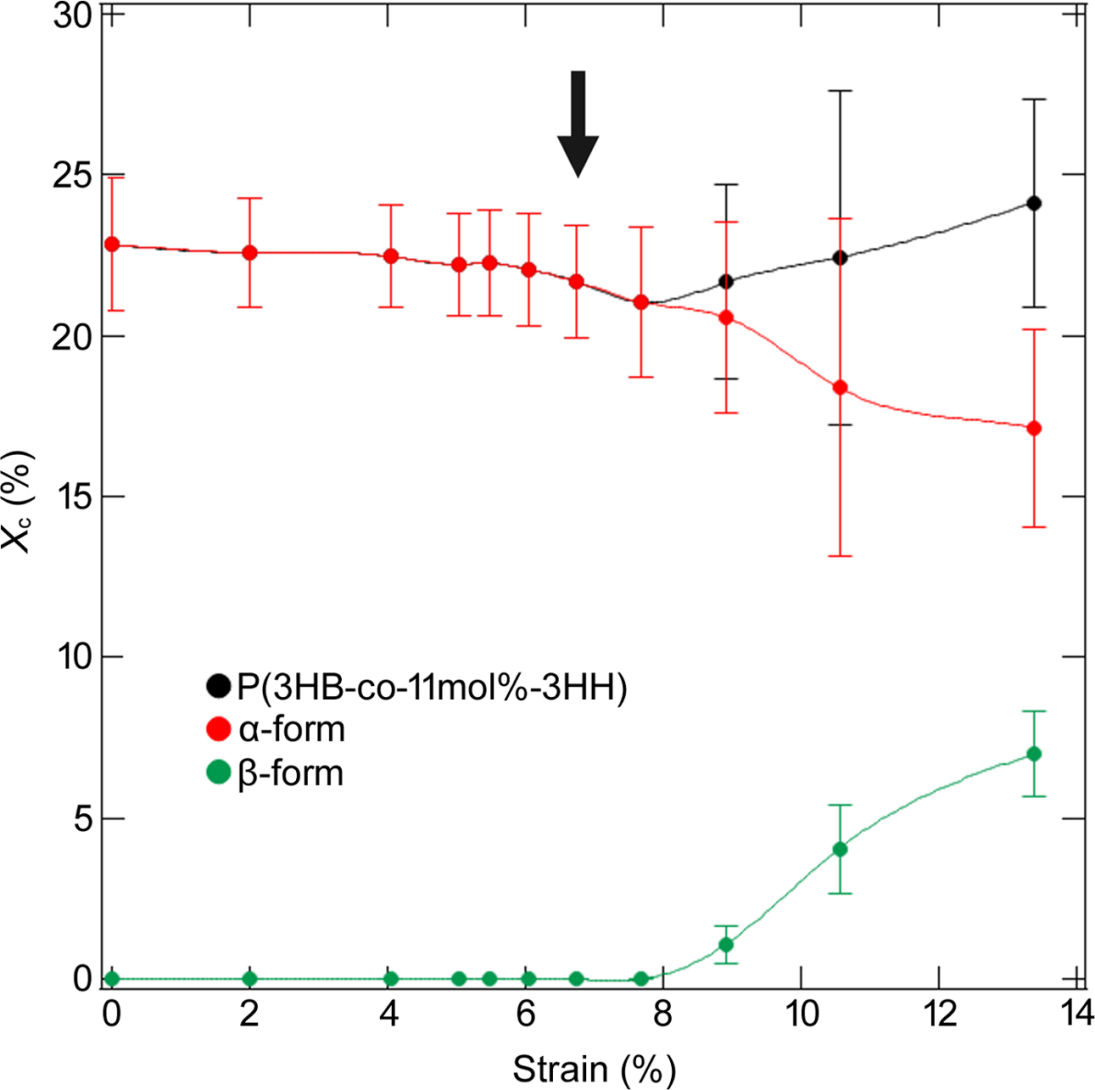
Crystallinity of P(3HB-*co*-11mol%-3HH) at each ratio. The black plot shows the transition of crystallinity of the entire P(3HB-*co*-11mol%-3HH) copolymer with strain, the red that of the α-form and the green that of the β-form. Arrow indicates the yield point.

The change in the crystallinity of the α-form (Iwata, 2005 ▸), as shown in Fig. 12 ▸, was different after ɛ = 6.73%, indicating that the rate of decrease in the crystallinity of the α-form became larger after ɛ = 6.73% and that mechanical melting occurs mainly after the yield point.

#### 3.4.2. Region II (7.68% ≤ ɛ)

In SAXS and USAXS, we observed streak scattering perpendicular to the stretching direction, as shown in Fig. 8 ▸. The appearance of streaks indicates that voids extended in the stretching direction were formed on length scales on the order of 10 nm to the submicrometre. In contrast to the homopolymer, the copolymer did not immediately fracture by stress dispersion due to copolymerization, even though nanovoids were generated and merged into submicrometre voids.

In region II, 〈*d*〉/〈*d*〉_0_ at μ = 90° could not be estimated from the correlation functions, indicating that the lamellar structures parallel to the stretching direction were deformed by coarse slip and that the stacking of the lamellae was disordered.

Regarding the Hermans orientation factor, the behaviors in region II were different from those in region I. The values of *f_a_*, *f_b_* and *f_c_* shifted closer to 0 under stretching, while the value of *f*_amo_ increased. After ɛ = 8.91%, the crystals appeared to be randomly oriented, but this reflected the information from the crystals where mechanical melting had not occurred, because mechanical melting occurred in the oriented crystals. The mechanical melting in the (020)_ortho_ plane of the α-form crystals oriented along the stretching direction is discussed in the supporting information.

We found an extra diffraction peak from the β-form in the direction perpendicular to the drawing direction after ɛ = 8.91%, as shown in Figs. 8 ▸(*q*) and 8 ▸(*r*). Orts *et al.* (1990 ▸) observed the formation of the β-phase in WAXD patterns when they stretched non-oriented poly(3-hy­droxy­butyrate-*c*o-3-hy­droxy­valerate). The β-form crystal is obtained by applying stress to some parts of the α-form lamellae and the amorphous components connected to these parts (Orts *et al.*, 1990 ▸; Iwata, 2005 ▸; Iwata *et al.*, 2005 ▸; Kabe *et al.*, 2016 ▸; Phongtamrug & Tashiro, 2019 ▸). Therefore, the WAXS results indicate that the molecular chains were pulled out from the (020)_ortho_ plane of the α-form crystals oriented along the stretching direction after ɛ = 8.91%, and then the pulled chains transformed into the β-form. As shown in Fig. 12 ▸, the increase in the crystallinity of the β-form was greater than the decrease in that of the α-form. This indicates that β-form crystals were formed not only by mechanical melting of α-form crystals but also by the chains in the amorphous phase.

### 3.5. Changes in the hierarchical structure and difference between P(3HB) and P(3HB-*co*-11mol%-3HH) under stretching

**Figure 13:**
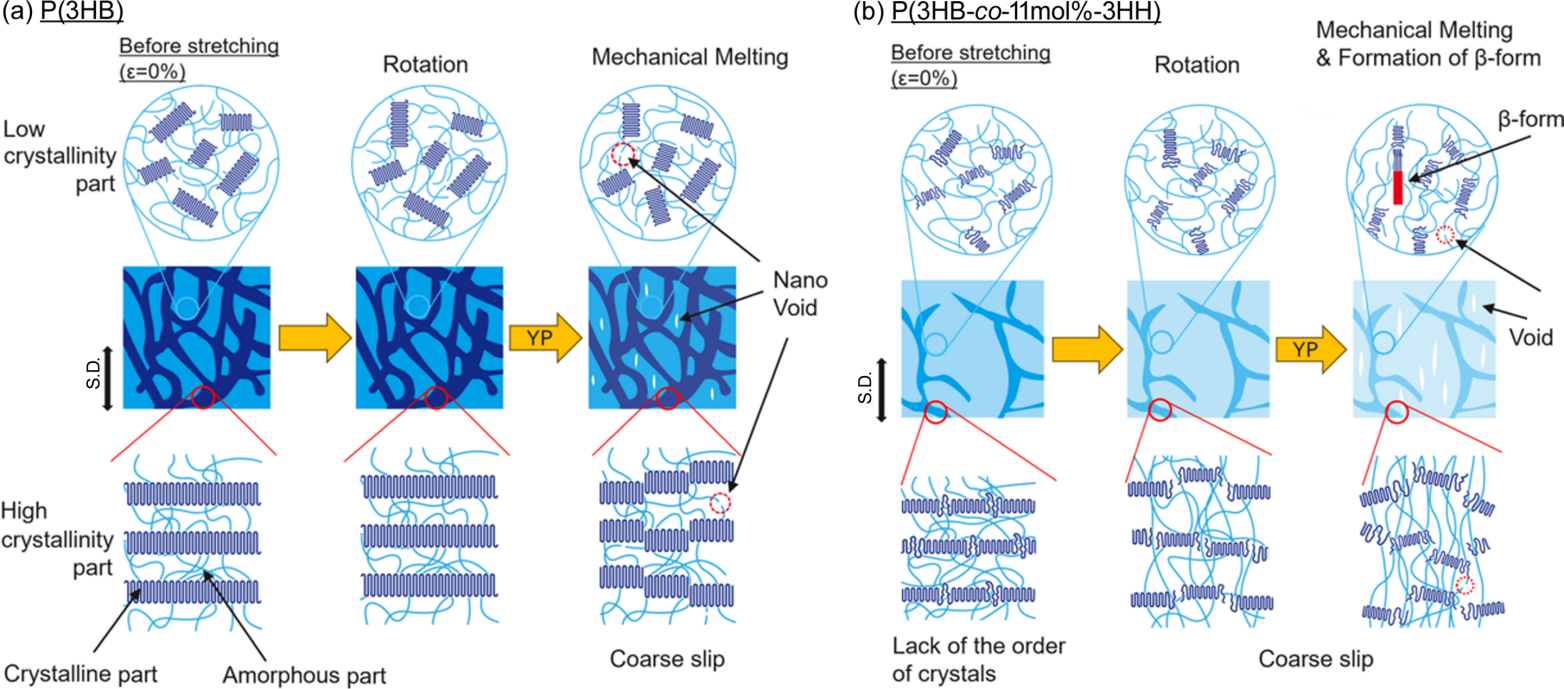
Illustrations of the changes in the hierarchical structure of (*a*) P(3HB) and (*b*) P(3HB-*co*-11mol%-3HH) during stretching

Fig. 13 ▸ shows illustrations of the changes in the hierarchical structure during stretching of the unoriented P(3HB) and P(3HB-*co*-11mol%-3HH), according to the above discussion.

P(3HB) consisted of thick crystals in lamellae and spherulite structures with a high regularity of lamellar stacking, resulting in high crystallinity. On the other hand, in P(3HB-*co*-11mol%-3HH), lamellae consisting of thin crystals were formed due to the introduction of the amorphous monomer units, and the crystallinity was lower. In addition, the co­poly­mer had poorer long-range order of the crystal structure than the homopolymer.

The copolymer and homopolymer showed similar orientation during stretching until the homopolymer broke. However, their deformation behaviors on the lamella to submicrometre scale were very different. The strong inhomogeneity characterized by the abnormal butterfly patterns was observed in the homopolymer on the fibril to submicrometre scale near the yield point, and the homopolymer ruptured soon after the creation of nanovoids.

On the other hand, the copolymer continued to deform beyond the elongation at which the homopolymer broke. Coarse slip occurred in the lamellae, and weak heterogeneous deformation on the submicrometre scale occurred near the yield point. This difference is attributed to the stability of crystal structures under strain relevant to the long-range order of crystal structures. The long-range order of crystals in the copolymer was poorer than that in the homopolymer, resulting in lower stress of the higher-crystallinity region or the fibrils for the copolymer compared with the homopolymer. Thus, the spatial heterogeneity of the stress field was lower in the copolymer and inhomogeneous deformation was less likely to occur. The low spatial inhomogeneity was also caused by the fragmentation of the lamellar structure due to the coarse slip. In addition, the coarse slip occurred possibly because of the thinning of the crystal thickness and the destabilization of crystals by copolymerization. The copoly­mer did not fracture due to stress dispersion even when voids formed on the nanometre to submicrometre scale after the yield point. Furthermore, after the yield point, mechanical melting occurred in the oriented crystals and the β-phases were formed. Since the *c* axis of the crystals was oriented perpendicular along the stretching direction without fracture, polymer chains were unfolded from the P(3HB) crystals, resulting in the formation of the β-form from the chains. The reason for the significant improvement in the ductility of the copolymer is mainly the dispersion of stress due to the introduction of P(3HH).

## 4. Conclusions

In this study, we investigated the structural changes of P(3HB) and P(3HB-*co*-11mol%-3HH) on various scales during uniaxial stretching by *in situ* USAXS, SAXS and WAXS. The P(3HB) homopolymer was highly crystallized and consisted of thick crystals in lamellae and spherulite structures with a high regularity of lamellar stacking. The introduction of amorphous monomer units suppressed crystallization, reduced long-range order of the crystal structure and formed thinner crystals in P(3HB-*co*-11mol%-3HH) than in the homopolymer.

Abnormal butterfly patterns, which characterize the heterogeneity of deformation on the submicrometre scale near the yield point, were observed in both samples by USAXS but were weaker for the copolymer. This is due to the stress dispersion caused by the hierarchical structure formed with the suppression of crystallization by copolymerization. Therefore, the effect of the introduction of P(3HH) is mainly the suppression of crystallization and the associated stress dispersion due to the hierarchical structure.

We have also clarified the mechanism of β-phase formation through mechanical melting while stretching unoriented P(3HB-*co*-11mol%-3HH).

In this study we examined samples under a specific thermal history. In a future study, we will investigate whether differences in hierarchical structure between homopolymers and copolymers under different crystallization conditions are consistent with the above result.

## Supplementary Material

Supporting figures and equations. DOI: 10.1107/S1600576725002365/ju5078sup1.pdf

## Figures and Tables

**Table 1:** Characteristic parameters before stretching (ɛ = 0%)

	*d*_l_ (nm)	*d*_c_ (nm)	*X*_c_ (%)
P(3HB)	7.36	5.39	61.70
P(3HB-*co*-11mol%-3HH)	7.50	2.14	22.82

## References

[bb1] Anderson, A. J. & Dawes, E. A. (1990). *Microbiol. Rev.* **54**, 450–472. 10.1128/mr.54.4.450-472.1990 PMC372789 2087222

[bb2] Bastide, J., Leibler, L. & Prost, J. (1990). *Macromolecules*, **23**, 1821–1825.

[bb3] Biddlestone, F., Harris, A., Hay, J. N. & Hammond, T. (1996). *Polym. Int.* **39**, 221–229.

[bb4] Butler, M. F., Donald, A. M. & Ryan, A. J. (1997). *Polymer*, **38**, 5521–5538.

[bb5] Doi, M. & Onuki, A. (1992). *J. Phys. II*, **2**, 1631–1656.

[bb6] Doi, Y., Kitamura, S. & Abe, H. (1995). *Macromolecules*, **28**, 4822–4828.

[bb7] Furukawa, A. & Tanaka, H. (2009). *Nat. Mater.* **8**, 601–609. 10.1038/nmat2468 19525951

[bb8] Gerasimov, V. I., Genin, Y. V. & Tsvankin, D. Y. (1974). *J. Polym. Sci. Polym. Phys. Ed.* **12**, 2035–2046.

[bb9] Guo, H., Wang, J., Zhou, C., Zhang, W., Wang, Z., Xu, B., Li, J., Shang, Y., de Claville Christiansen, J., Yu, D., Wu, Z. & Jiang, S. (2015). *Polymer*, **70**, 109–117.

[bb10] Hashimoto, T. (2022). *Principles and applications of X-ray, light and neutron scattering.* Springer Nature Singapore Pte Ltd.

[bb11] Hayes, C., Bokobza, L., Boué, F., Mendes, E. & Monnerie, L. (1996). *Macromolecules*, **29**, 5036–5041.

[bb12] Iwata, T. (2005). *Macromol. Biosci.* **5**, 689–701. 10.1002/mabi.200500066 16052600

[bb13] Iwata, T., Fujita, M., Aoyagi, Y., Doi, Y. & Fujisawa, T. (2005). *Biomacromolecules*, **6**, 1803–1809. 10.1021/bm050152s 15877408

[bb14] Kabe, T., Tanaka, T., Marubayashi, H., Hikima, T., Takata, M. & Iwata, T. (2016). *Polymer*, **93**, 181–188.

[bb15] Karino, T., Okumura, Y., Zhao, C., Kataoka, T., Ito, K. & Shibayama, M. (2005). *Macromolecules*, **38**, 6161–6167.

[bb16] Kishimoto, M., Mita, K., Ogawa, H. & Takenaka, M. (2020). *Macromolecules*, **53**, 9097–9107.

[bb17] Koning, G. J. M. de & Lemstra, P. J. (1992). *Polymer*, **33**, 3292–3294.

[bb18] Koning, G. J. M. de & Lemstra, P. J. (1993). *Polymer*, **34**, 4089–4094.

[bb19] Kurotani, Y. & Tanaka, H. (2022). *Commun. Mater.* **3**, 67.

[bb20] Liebergesell, M., Mayer, F. & Steinbüchel, A. (1993). *Appl. Microbiol. Biotechnol.* **40**, 292–300.

[bb21] Masunaga, H., Ogawa, H., Takano, T., Sasaki, S., Goto, S., Tanaka, T., Seike, T., Takahashi, S., Takeshita, K., Nariyama, N., Ohashi, H., Ohata, T., Furukawa, Y., Matsushita, T., Ishizawa, Y., Yagi, N., Takata, M., Kitamura, H., Sakurai, K., Tashiro, K., Takahara, A., Amamiya, Y., Horie, K., Takenaka, M., Kanaya, T., Jinnai, H., Okuda, H., Akiba, I., Takahashi, I., Yamamoto, K., Hikosaka, M., Sakurai, S., Shinohara, Y., Okada, A. & Sugihara, Y. (2011). *Polym. J.* **43**, 471–477.

[bb22] Orts, W. J., Marchessault, R. H., Bluhm, T. L. & Hamer, G. K. (1990). *Macromolecules*, **23**, 5368–5370.

[bb23] Phongtamrug, S. & Tashiro, K. (2019). *Macromolecules*, **52**, 2995–3009.

[bb24] Sakurai, T., Nozue, Y., Kasahara, T., Mizunuma, K., Yamaguchi, N., Tashiro, K. & Amemiya, Y. (2005). *Polymer*, **46**, 8846–8858.

[bb25] Strobl, G. R. & Schneider, M. (1980). *J. Polym. Sci. Polym. Phys. Ed.* **18**, 1343–1359.

[bb26] Strobl, G. R., Schneider, M. J. & Voigt–Martin, I. G. (1980). *J. Polym. Sci. Polym. Phys. Ed.* **18**, 1361–1381.

[bb27] Sudesh, K., Abe, H. & Doi, Y. (2000). *Prog. Polym. Sci.* **25**, 1503–1555.

[bb28] Takenaka, M., Shimizu, H. & Nishitsuji, S. (2007). *Phys. Rev. E*, **75**, 061802. 10.1103/PhysRevE.75.061802 17677289

[bb29] Wilchinsky, Z. W. (1959). *J. Appl. Phys.* **30**, 792.

[bb30] Wilchinsky, Z. W. (1960). *J. Appl. Phys.* **31**, 1969–1972.

