# Differences in hierarchical structural changes between unoriented P(3HB) and P(3HB-*co*-3HH) under stretching. Corrigendum

**DOI:** 10.1107/S1600576726000725

**Published:** 2026-02-01

**Authors:** Masato Arakawa, Taizo Kabe, Tadahisa Iwata, Mikihito Takenaka

**Affiliations:** a Institute for Chemical Research Kyoto University Gokasho Uji Kyoto611-0011 Japan; b Science of Polymeric Materials, Department of Biomaterial Sciences, Graduate School of Agricultural and Life Sciences University of Tokyo 1-1-1 Yayoi Bunkyo-Ku Tokyo113-8657 Japan

**Keywords:** P(3HB), P(3HB-*co*-3HH), crystalline polymers, USAXS, ultra-small-angle X-ray scattering, stretch-induced density fluctuations, *in situ*X-ray scattering

## Abstract

The funding information for the article by Arakawa *et al.* [*J. Appl. Cryst.* (2025), **58**, 886–896] is corrected.

In the article by Arakawa *et al.* (2025 ▸), the funding information was incorrectly given. The corrected information is given here.
