# Supplementary material for: Differences in hierarchical structural changes between unoriented P(3HB) and P(3HB-co-3HH) under stretching
Source: J Appl Crystallogr. 2025 Apr 25;58(Pt 3):886–96. doi: 10.1107/S1600576725002365 (PMC12135995; doi:10.1107/S1600576725002365)
Supplement: Supplementary file 1 [file j-58-00886-sup1.pdf]

JOURNAL OF APPLIED CRYSTALLOGRAPHY

Volume 58 (2025)

Supporting information for article:

Differences in hierarchical structural changes between unoriented P(3HB) and P(3HB-*co*-3HH) under stretching

Masato Arakawa, Taizo Kabe, Tadahisa Iwata and Mikihito Takenaka

S1. Estimation of the average d-spacing during uniaxial stretching and crystal thickness

To investigate the changes in lamellar structure caused by stretching in more detail, we performed the following analyses. Firstly, we obtained the SAXS 1D profiles by averaging sectors in 2D patterns at every 5˚ from μ = 0˚ to 90˚, and then the profiles at each azimuthal angle μ were smoothed by binomial smoothing in Igor® software. To remove the overlap of the butterfly patterns to the peaks from the lamellae, we subtracted the Debye-Bueche scattering function fitted on the low-q region from the smoothed SAXS profiles from μ = 20˚ to 90˚ after ε = 1.8% in the homopolymer. Secondary, the correlation functions corresponding to each μ were calculated from the smoothed profiles, respectively. Correlation functions K(z) at length z from sector averages at every 5˚ in the μ range from 0˚ to 90˚ from the SAXS 2D patterns is defined as

$$K(z) = \frac{\int_{0.21\,nm^{-1}}^{1.93\,nm^{-1}} q^2 I(q) \cos qz \ \mathrm{d}q}{\int_{0.21\,nm^{-1}}^{1.93\,nm^{-1}} q^2 I(q) \ \mathrm{d}q} \qquad (S1)$$

According to a book chapter (Hashimoto, 2022), the 'Lorentz correction' can be applied to grains containing lamellae with arbitrary orientation distribution in 3-D space. In the reference, it is written that the scattered intensity is multiplied by a constant given by a probability that the principal axis of the grain has the orientation parallel to the scattering vector $\boldsymbol{q}$. Thus, we can apply Eq. S1 to stretched samples with anisotropic lamellar orientation distributions.

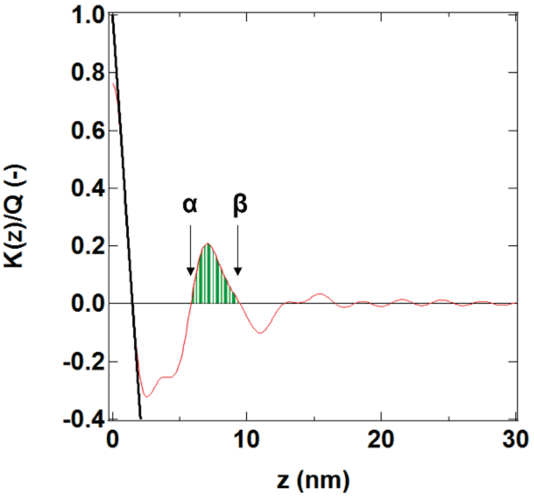

Figure S1 The correlation function K(z) obtained from the 1D profile of unstretched P(3HB). This function is normalized by Q which is the intersection of the K(z) axis and the extrapolation of the linear part in the K(z).

Figure S1 shows an example of $K(z)$ from the 1D profile of unstretched P(3HB). The averages of d-spacing $\langle d \rangle$ of lamellar structures are given by

$$\langle d \rangle = \frac{\int_{\alpha}^{\beta} z\, K(z)\, \mathrm{dz}}{\int_{\alpha}^{\beta} K(z)\, \mathrm{dz}} \qquad (S2)$$

where α is the z at which K(z) is positive the second time when z is increased from 0, and β is the z at which K(z) is negative the second time, as shown in Figure S1.

The average crystal thickness is estimated by the intersection of the K(z) minimum and the extrapolation of the linear part in the K(z) in Figure S1.

S2. Estimation of the average Hermans orientation factor from WAXS

The Hermans orientation factor is defined as

$$f_H = \frac{3\langle \cos^2 \emptyset_i \rangle - 1}{2} \qquad (S3)$$

where $\emptyset_i$ is the angle between the unit within a crystal of interest (e.g., c-axis) and the stretching direction. Assuming a rotational symmetry around the stretching direction,

$$\langle \cos^2 \emptyset_i \rangle = \frac{\int_0^{\frac{\pi}{2}} I(\emptyset_i) \cos^2 \emptyset_i \sin \emptyset_i \, d\emptyset_i}{\int_0^{\frac{\pi}{2}} I(\emptyset_i) \sin \emptyset_i \, d\emptyset_i} \qquad (S4)$$

where $I(\emptyset_i)$ is the pole concentration corresponding to the relative amount of crystalline material having plane normals in the direction of $\emptyset_i$. We obtained orientation information for each axis of crystals and amorphous polymer chains by using Wilchinsky's method (1959, 1960);

$$\langle \cos^2 \emptyset_{b-axis} \rangle = \langle \cos^2 \emptyset_{(020)_{ortho}} \rangle \qquad (S5)$$

$$\langle \cos^2 \emptyset_{c-axis} \rangle = 1 - 0.810 \langle \cos^2 \emptyset_{(020)_{ortho}} \rangle - 1.190 \langle \cos^2 \emptyset_{(110)_{ortho}} \rangle \qquad (S6)$$

$$\langle \cos^2 \emptyset_{a-axis} \rangle + \langle \cos^2 \emptyset_{b-axis} \rangle + \langle \cos^2 \emptyset_{c-axis} \rangle = 1 \qquad (S7)$$

$$f_a = \frac{3\langle \cos^2 \emptyset_{a-axis} \rangle - 1}{2}, f_b = \frac{3\langle \cos^2 \emptyset_{b-axis} \rangle - 1}{2}, f_c = \frac{3\langle \cos^2 \emptyset_{c-axis} \rangle - 1}{2} \qquad (S8)$$

$$f_{H,amo} = \frac{3\langle \cos^2 \emptyset_{amo} \rangle - 1}{2} \qquad (S9)$$

S3. Estimation of the crystallinity during uniaxial stretching

The crystallinities of P(3HB) and P(3HB-*co*-11mol%-3HH) during uniaxial stretching, $X_c(\varepsilon)$, are given by

$$X_c(\varepsilon) = \frac{W_c(\varepsilon)}{W_c(\varepsilon) + W_a(\varepsilon)} \quad (S10)$$

where $W_c(\varepsilon)$, and $W_a(\varepsilon)$ are the weight of the crystalline and amorphous phases at ε, respectively. We averaged sectors at every 5° in the μ range from 180° to 270° from the WAXS 2D patterns to calculate WAXS 1D profiles and obtained these terms by separating those profiles into peaks originating from the crystalline phase and peaks originating from the amorphous phase by combining Gaussian functions and a constant (background). Assuming that the scattering intensity distribution is symmetrical along the stretching axis, $W_c(\varepsilon)$, and $W_a(\varepsilon)$ can be defined by the following equations.

$$W_c(\varepsilon) = 4\pi \sum_{i=1}^{7} \int_{\pi}^{\frac{3}{2}\pi} q_{\mathrm{c},i}(\varepsilon,\mu) I_{\mathrm{c},i}(\varepsilon,\mu) w_{\mathrm{c},i}(\varepsilon,\mu) \sin\mu \, d\mu \quad (S11)$$

$$W_a(\varepsilon) = 4\pi \int_{\pi}^{\frac{3}{2}\pi} q_{\mathrm{a}}(\varepsilon,\mu) I_{\mathrm{a}}(\varepsilon,\mu) w_{\mathrm{a}}(\varepsilon,\mu) \sin\mu \, d\mu \quad (S12)$$

where q_{c}, I_{c} and w_{c} are, respectively, the position, intensity, and width of the crystalline peak on the profiles, and q_{a}, I_{a} and w_{a} are of the amorphous halo. The crystalline peaks numbered i = 1-5 correspond to the reflections from $(020)_{\mathrm{ortho}}$, $(011)_{\mathrm{ortho}}$, $(110)_{\mathrm{ortho}}$, $(021)_{\mathrm{ortho}}$, $(101)_{\mathrm{ortho}}$, $(111)_{\mathrm{ortho}}$ planes, and β-form, respectively.

S4. USAXS, SAXS and WAXS 2D patterns for the P(3HB-*co*-11mol%-3HH) in region Ⅰ and Ⅱ

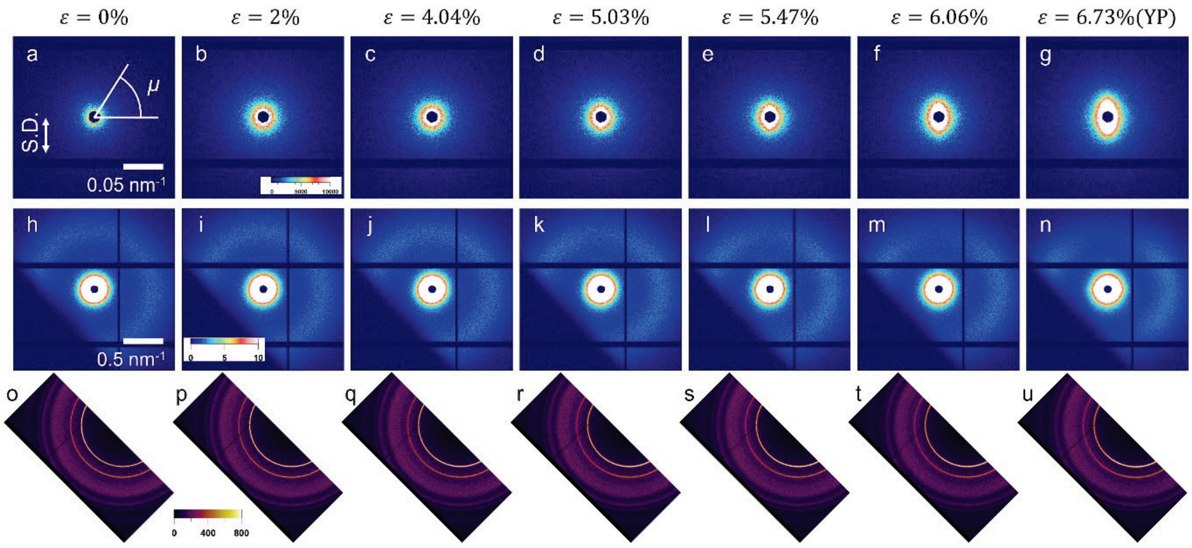

Figure S2 (a–g) USAXS, (h–n) SAXS, and (o–u) WAXS 2D patterns of the P(3HB-*co*-11mol%-3HH) with strain in region I. The stretching direction (S.D.) is parallel along the arrow in (a).

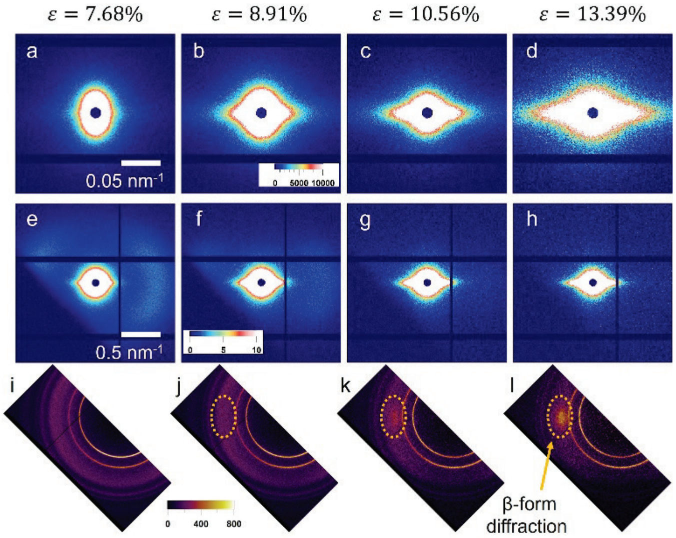

Figure S3 (a–d) USAXS, (e–h) SAXS, and (i–l) WAXS 2D patterns of the P(3HB-*co*-11mol%-3HH) with strain in region II. The stretching direction (S.D.) is parallel along the arrow in (a).

Figure S2 and S3 shows the changes in the USAXS, SAXS, and WAXS 2D patterns with strain for the P(3HB-*co*-11mol%-3HH).

S5. The μ dependence of full width at half maximum (FWHM) of the diffraction peaks in WAXS 2D patterns for the P(3HB)

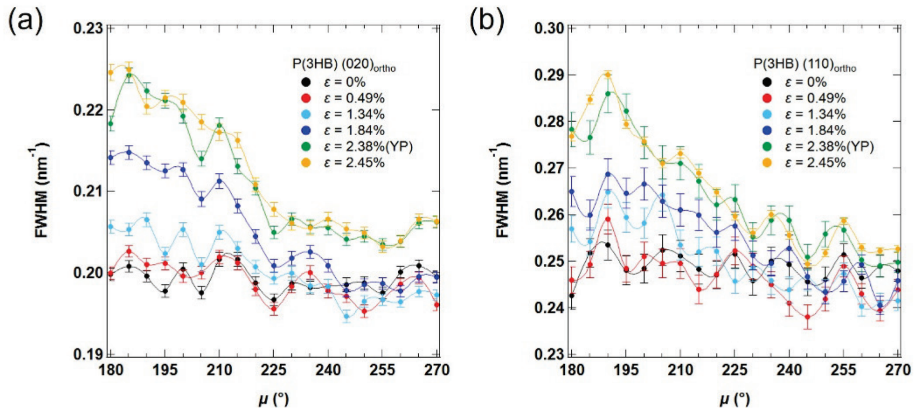

Figure S4 μ dependence of FWHM of the diffraction peaks from (a) $(020)_{ortho}$ and (b) $(110)_{ortho}$ lattice plane for the P(3HB) with strain.

Figure S4 shows the μ dependence of FWHM of the diffraction peaks from $(020)_{ortho}$ and $(110)_{ortho}$ lattice planes in the orthorhombic phase in WAXS 2D patterns for the P(3HB), respectively. According to Figure S4, the FWHM of the $(020)_{ortho}$ and $(110)_{ortho}$ peaks around $\mu = 180^{\circ}$ increased with strain. This indicates that as the stretch ratio increased, the alignments of these lattice planes along the stretch direction were disordered. In other words, the lamellar structures parallel to the stretch direction collapsed.

S6. The μ dependence of the diffraction peaks in WAXS 2D patterns for the P(3HB-*co*-11mol%-3HH)

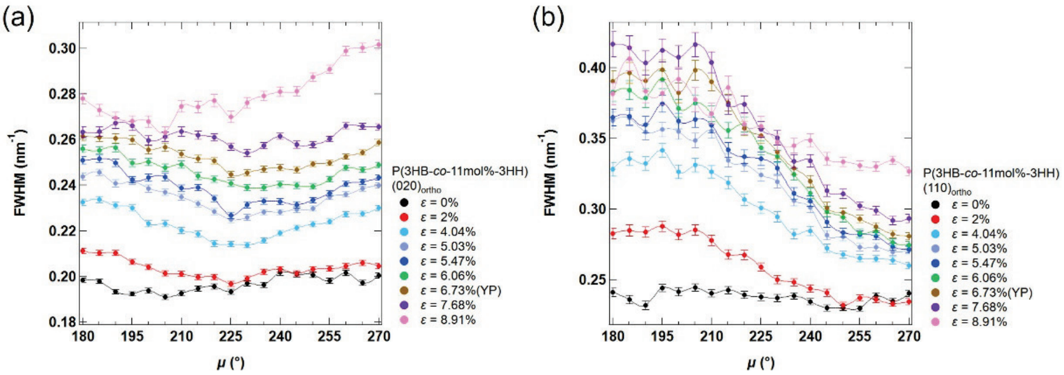

Figure S5 μ dependence of FWHM of the diffraction peaks from (a) $(020)_{ortho}$ and (b) $(110)_{ortho}$ lattice plane for the P(3HB-*co*-11mol%-3HH) with strain.

Figure S5(a) shows the FWHM of $(020)_{ortho}$ peak at all azimuthal angles increased with strain. Unlike the homopolymer, the alignments of the $(020)_{ortho}$ plane were disordered without depending on the azimuthal angles by stretch, indicating that the introduction of amorphous component units reduced the stability of the crystals.

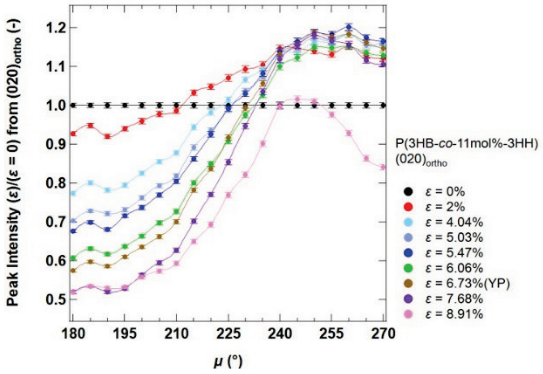

Figure S6 μ dependence of the peak intensity normalized by that at $\varepsilon = 0\%$ from $(020)_{ortho}$ plane for the P(3HB-*co*-11mol%-3HH).

On the other hand, the FWHM of the $(110)_{ortho}$ peak around $\mu = 180^\circ$ increased as with the homopolymer as shown in Figure S5(b). Considering the disappearance of the SAXS peaks along $\mu = 90^\circ$ after $\varepsilon = 5.03\%$, the coarse slip occurred on the $(110)_{ortho}$ plane because of the μ dependence of FWHM of the $(110)_{ortho}$ peak.

Figure S6 shows diffraction peak intensity at each μ from $(020)_{ortho}$ plane in WAXS 2D patterns for the copolymer normalized by that at $\varepsilon = 0\%$. A decrease in the intensity around $\mu = 270^\circ$ at $\varepsilon = 8.91\%$ indicates that mechanical melting, that is, unfolding of the α-form crystal occurred in $(020)_{ortho}$ plane along the stretching direction.
